# Crystal structure of 2-{(*E*)-[(2-hy­droxy­phen­yl)iminium­yl]meth­yl}-4-methyl­phenolate

**DOI:** 10.1107/S2056989015006374

**Published:** 2015-04-09

**Authors:** Suresh Shalini, C. R. Girija, Mukesh M. Jotani, C. D. Sathish, T. V. Venkatesha

**Affiliations:** a Chemistry Research Centre (Affiliated to Kuvempu University), SSMRV Degree College, Jayanagar 4th T Block, Bangalore 560 041, Karnataka, India; b Govt. Science College, Nrupatunga Road, Ambedkar Veedhi, Sampangi Rama Nagar, Bengaluru 560 001, Karnataka, India; c Department of Physics, Bhavans Sheth R. A. College of Science, Khanpur, Ahmedabad 380 001, Gujarat, India; d Department of Chemistry, PESIT, BSK III Stage, Bangalore 560 085, India; e Department of Chemistry, Jnana Sahyadri, Kuvempu University, Shankaragatta 577 451, India

**Keywords:** crystal structure, Schiff base, *N*-(salicyl­idene)aniline, zwitterion, hydrogen bonding

## Abstract

The title compound, C_14_H_13_NO_2_, exists as a zwitterion in the solid state, with the H atom of the phenol group transferred to the imine N atom. The dihedral angle between the planes of the benzene rings is 10.13 (9)°. Intra­molecular N—H⋯O hydrogen bond generate *S*(6) and *S*(5) loops. In the crystal, mol­ecules are connected by O—H⋯O hydrogen bonds, generating *C*(9) chains propagating in the [010] direction.

## Related literature   

For a related structure, see: Eltayeb *et al.* (2010 ▸). For background to Schiff bases and their applications, see: Blagus *et al.* (2010 ▸). 
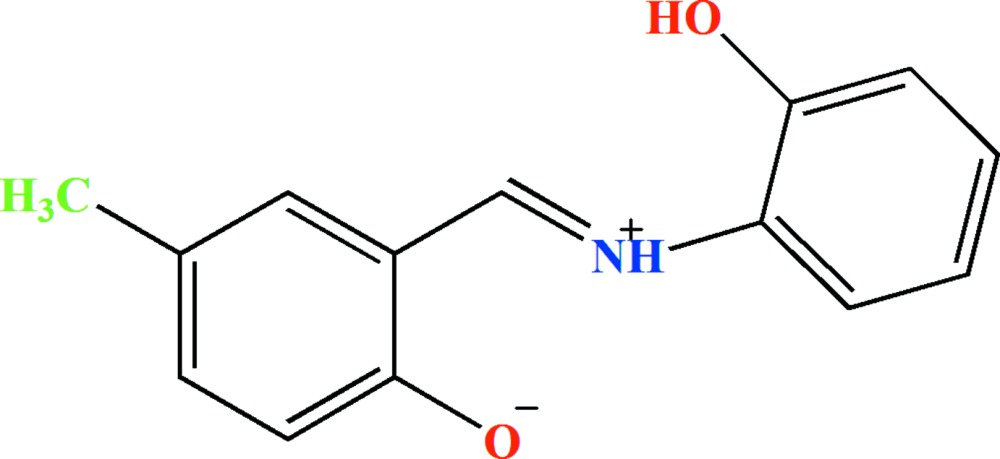


## Experimental   

### Crystal data  

C_14_H_13_NO_2_
*M* *_r_* = 227.25
Orthorhombic, 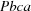

*a* = 12.9474 (18) Å
*b* = 9.0660 (13) Å
*c* = 19.583 (3) Å
*V* = 2298.7 (6) Å^3^
*Z* = 8
Mo *K*α radiation
μ = 0.09 mm^−1^
*T* = 293 K
0.30 × 0.25 × 0.20 mm

### Data collection  

Bruker APEXII CCD diffractometer
Absorption correction: multi-scan (*SADABS*; Bruker, 2009 ▸) *T*_min_ = 0.875, *T*_max_ = 1.000
29481 measured reflections
2583 independent reflections
1810 reflections with *I* > 2σ(*I*)
*R*_int_ = 0.059

### Refinement  

*R*[*F* ^2^ > 2σ(*F* ^2^)] = 0.050
*wR*(*F* ^2^) = 0.130
*S* = 1.05
2583 reflections
163 parameters
2 restraints
H atoms treated by a mixture of independent and constrained refinement
Δρ_max_ = 0.21 e Å^−3^
Δρ_min_ = −0.20 e Å^−3^

### 

Data collection: *APEX2* (Bruker, 2009 ▸); cell refinement: *SAINT* (Bruker, 2009 ▸); data reduction: *SAINT*; program(s) used to solve structure: *SHELXS97* (Sheldrick, 2008 ▸); program(s) used to refine structure: *SHELXL2014* (Sheldrick, 2015 ▸); molecular graphics: *ORTEP-3 for Windows* (Farrugia, 2012 ▸) and *PLATON* (Spek, 2009 ▸); software used to prepare material for publication: *SHELXL2014* and *PLATON*.

## Supplementary Material

Crystal structure: contains datablock(s) I. DOI: 10.1107/S2056989015006374/hb7370sup1.cif

Structure factors: contains datablock(s) I. DOI: 10.1107/S2056989015006374/hb7370Isup2.hkl

**Fig. 1.**
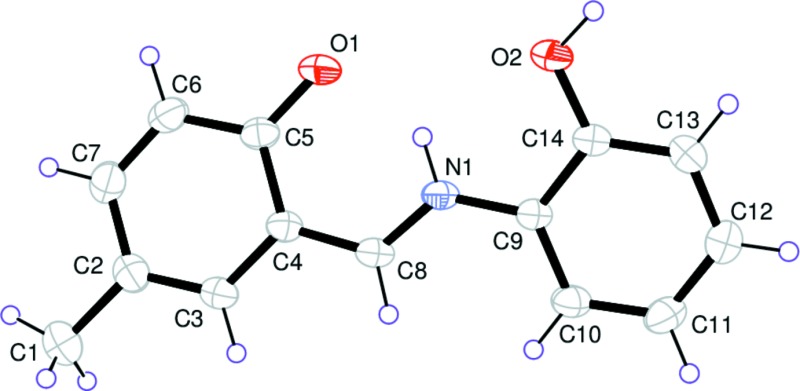
ORTEP Plot of (I) drawn at 40% probability level

ORTEP . DOI: 10.1107/S2056989015006374/hb7370fig1.tif
*ORTEP* Plot of (I) drawn at 40% probability level

**Fig. 2.**
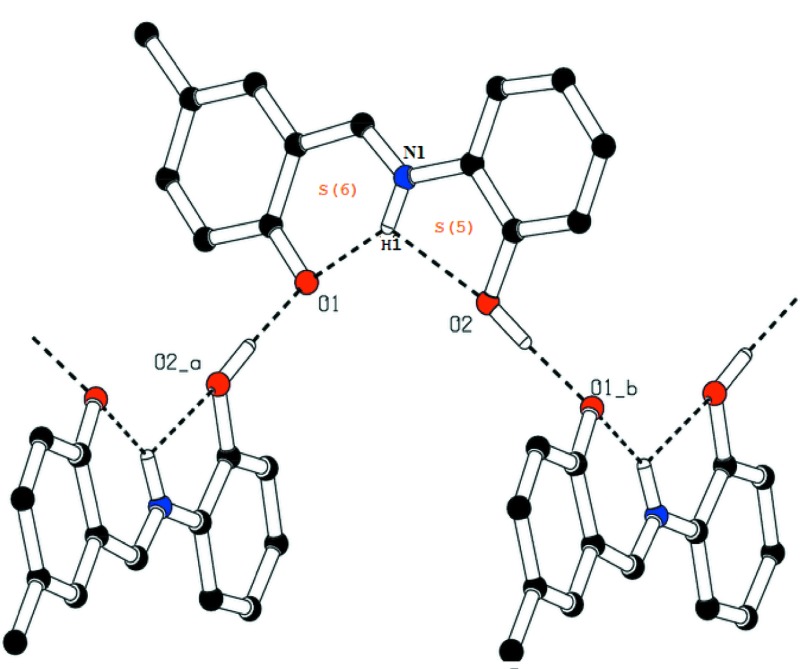
A perspective view of the one-dimensional infinite chain in the title compound, (I), showing N—H···O and O—H···O hydrogen-bnd interactions as dashed lines. H atoms not involved in the interactions have been omitted for the sake of clarity.

. DOI: 10.1107/S2056989015006374/hb7370fig2.tif
A perspective view of the one-dimensional infinite chain in the title compound, (I), showing N—H⋯O and O—H⋯O hydrogen-bnd inter­actions as dashed lines. H atoms not involved in the inter­actions have been omitted for the sake of clarity.

CCDC reference: 1005919

Additional supporting information:  crystallographic information; 3D view; checkCIF report

## Figures and Tables

**Table 1:** Hydrogen-bond geometry (, )

*D*H*A*	*D*H	H*A*	*D* *A*	*D*H*A*
O2H4O1^i^	0.93(2)	1.65(2)	2.5756(18)	176(3)
N1H1O2	0.90(2)	2.32(2)	2.6598(19)	102(2)
N1H1O1	0.90(2)	1.84(2)	2.5933(19)	141(2)

Symmetry code: (i) 
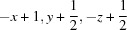
.

## supplementary crystallographic information

### S1. Chemical context

N-substituted imines, also known as Schiff bases represent one of the most widely used families of organic compounds. Schiff bases have been intensively used as synthetic inter­mediates and as ligands for coordinating transition and inner transition metal ions, and recently also for coordinating anions. Schiff base ligands may contain a variety of substituents with different electron-donating or electron-withdrawing groups, and therefore may have inter­esting chemical properties. They have attracted particular inter­est due to their biological activities acting as radiopharmaceuticals for cancer targeting.They have also been used as model systems for biological macromolecules . Besides the biological activity, solid-state thermochromism and photochromism are an another characteristic of these compounds leading to their application in various areas of materials science such as the control and measurement of radiation intensity, display systems and optical memory devices . Schiff bases derived from o-hy­droxy­aromatic aldehydes and ketones are excellent models for the study of keto-enol tautomerism both in solution and in the solid state (Blagus *et al.*, 2010).

### S2. Structural commentary

The structure of the title compound is as shown in Fig.1 is described in terms of three planar subunits,namely two terminal benzene rings and their substituents bridged by a C=N moiety. The molecule has adopted E-configuration about the C8—N1 double bond (1.301 (2)Å) with a C9—N1—C8—C4 torsion angle of 179.90 (16)°. The C4—C8 and N1—C9 bond distances [1.410 (2) and 1.404 (2)Å] confirm π-electron delocalisation between the phenyl rings. The N1—C8—C4[123.26 (15)°] is greater than the normal value of 120°. This may be due to inter­action of iminium H with phenolate O atom. The C6—C5—C4 [116.51 (15)°] is smaller than the normal value of 120° which is due to lengthening of the phenolate C5—O1 [1.304 (2)Å] bond. All other bond distances and bond angles are within the normal range (Eltayeb *et al.*, 2010).

### S3. Supra­molecular features

The iminium H atom is engaged in a strong intra­molecular hydrogen bond with the O atom of the phenolate (N^+^ —H···O ) to form a S(6) motif. The crystal structure is stabilised by both intra­molecular N1—H1···O1 and inter­molecular O2—H4···O1 hydrogen bonding linking the molecules into infinite one-dimensional chains as shown in the figure.2, table.2, extending along the b-axis of the unit cell.

### S4. Synthesis and crystallization

*o*-Amino­phenol (5.45g, 0.01mole) was taken in 100mL round bottom flask. Salicyl­aldehyde (6.10g, 0.01mole) was added to the round bottom flask in methanol medium. The resulting mixture was refluxed for about 30 min. The resulting Schiff base was separated as orange crystals. The product was filtered, washed and recrystallized from methanol (M.P.134-135 ^0^C, Yield 75%). Single crystals of the compound were grown by slow evaporation method using ethanol as solvent at room temperature.

### S5. Refinement

Crystal data, data collection and structure refinement details are summarized in Table 1.

### Crystal data

**Table:**

C_14_H_13_NO_2_	*D*_x_ = 1.313 Mg m^−^^3^
*M**_r_* = 227.25	Melting point: 355 K
Orthorhombic, *P**b**c**a*	Mo *K*α radiation, λ = 0.71073 Å
*a* = 12.9474 (18) Å	Cell parameters from 500 reflections
*b* = 9.0660 (13) Å	θ = 5.0–50.0°
*c* = 19.583 (3) Å	µ = 0.09 mm^−^^1^
*V* = 2298.7 (6) Å^3^	*T* = 293 K
*Z* = 8	Block, colorless
*F*(000) = 960	0.3 × 0.25 × 0.20 mm

### Data collection

**Table:**

Bruker APEXII CCD diffractometer	1810 reflections with *I* > 2σ(*I*)
Radiation source: graphite monochromator	*R*_int_ = 0.059
OMEGA–PHI scans	θ_max_ = 27.6°, θ_min_ = 2.6°
Absorption correction: multi-scan (*SADABS*; Bruker, 2009)	*h* = −16→16
*T*_min_ = 0.875, *T*_max_ = 1.000	*k* = −11→8
29481 measured reflections	*l* = −25→25
2583 independent reflections	

### Refinement

**Table:**

Refinement on *F*^2^	2 restraints
Least-squares matrix: full	Hydrogen site location: mixed
*R*[*F*^2^ > 2σ(*F*^2^)] = 0.050	H atoms treated by a mixture of independent and constrained refinement
*wR*(*F*^2^) = 0.130	*w* = 1/[σ^2^(*F*_o_^2^) + (0.0467*P*)^2^ + 1.0454*P*] where *P* = (*F*_o_^2^ + 2*F*_c_^2^)/3
*S* = 1.05	(Δ/σ)_max_ = 0.015
2583 reflections	Δρ_max_ = 0.21 e Å^−^^3^
163 parameters	Δρ_min_ = −0.20 e Å^−^^3^

### Special details

**Table:**

Geometry. All e.s.d.'s (except the e.s.d. in the dihedral angle between two l.s. planes) are estimated using the full covariance matrix. The cell e.s.d.'s are taken into account individually in the estimation of e.s.d.'s in distances, angles and torsion angles; correlations between e.s.d.'s in cell parameters are only used when they are defined by crystal symmetry. An approximate (isotropic) treatment of cell e.s.d.'s is used for estimating e.s.d.'s involving l.s. planes.

### Fractional atomic coordinates and isotropic or equivalent isotropic displacement parameters (Å^2^)

**Table:**

	*x*	*y*	*z*	*U*_iso_*/*U*_eq_	
H1	0.6541 (13)	0.138 (3)	0.2650 (11)	0.063 (7)*	
H4	0.5002 (18)	0.378 (3)	0.1982 (14)	0.099 (9)*	
N1	0.71804 (10)	0.16956 (16)	0.25537 (7)	0.0328 (3)	
C8	0.78912 (12)	0.1022 (2)	0.29051 (8)	0.0345 (4)	
H8	0.8579	0.1254	0.2819	0.041*	
O1	0.58630 (9)	0.00525 (16)	0.31990 (7)	0.0465 (4)	
C9	0.73191 (11)	0.2767 (2)	0.20440 (8)	0.0315 (4)	
O2	0.55197 (9)	0.31121 (17)	0.20963 (7)	0.0494 (4)	
C14	0.64308 (12)	0.3480 (2)	0.18036 (9)	0.0350 (4)	
C5	0.66350 (13)	−0.0477 (2)	0.35492 (9)	0.0358 (4)	
C10	0.82779 (13)	0.3143 (2)	0.17751 (9)	0.0389 (4)	
H10	0.8872	0.2685	0.1937	0.047*	
C4	0.76708 (12)	−0.0042 (2)	0.34097 (8)	0.0335 (4)	
C2	0.83378 (15)	−0.1684 (2)	0.42880 (10)	0.0451 (5)	
C13	0.65167 (14)	0.4516 (2)	0.12897 (9)	0.0415 (5)	
H13	0.5928	0.4982	0.1124	0.050*	
C6	0.64951 (14)	−0.1513 (2)	0.40786 (9)	0.0441 (5)	
H6	0.5830	−0.1815	0.4191	0.053*	
C11	0.83474 (14)	0.4189 (2)	0.12694 (10)	0.0462 (5)	
H11	0.8990	0.4445	0.1094	0.055*	
C3	0.84905 (13)	−0.0673 (2)	0.37804 (9)	0.0418 (5)	
H3	0.9163	−0.0390	0.3676	0.050*	
C7	0.73159 (15)	−0.2085 (2)	0.44311 (9)	0.0461 (5)	
H7	0.7190	−0.2763	0.4778	0.055*	
C12	0.74692 (16)	0.4864 (2)	0.10209 (9)	0.0457 (5)	
H12	0.7520	0.5554	0.0671	0.055*	
C1	0.92146 (18)	−0.2371 (3)	0.46830 (12)	0.0691 (7)	
H1A	0.9742	−0.2687	0.4372	0.104*	
H1B	0.8963	−0.3205	0.4935	0.104*	
H1C	0.9497	−0.1658	0.4994	0.104*	

### Atomic displacement parameters (Å^2^)

**Table:**

	*U*^11^	*U*^22^	*U*^33^	*U*^12^	*U*^13^	*U*^23^
N1	0.0232 (6)	0.0327 (9)	0.0425 (8)	−0.0010 (6)	−0.0004 (6)	−0.0013 (6)
C8	0.0243 (7)	0.0355 (11)	0.0437 (9)	−0.0001 (7)	−0.0016 (7)	−0.0035 (8)
O1	0.0253 (6)	0.0571 (10)	0.0572 (8)	−0.0049 (6)	−0.0043 (5)	0.0051 (7)
C9	0.0267 (8)	0.0304 (10)	0.0372 (8)	−0.0001 (7)	−0.0005 (6)	−0.0027 (7)
O2	0.0249 (6)	0.0582 (10)	0.0651 (9)	0.0050 (6)	0.0004 (6)	0.0148 (7)
C14	0.0271 (8)	0.0381 (11)	0.0398 (9)	0.0005 (7)	−0.0008 (7)	−0.0029 (8)
C5	0.0305 (8)	0.0360 (11)	0.0410 (9)	−0.0041 (7)	0.0000 (7)	−0.0059 (8)
C10	0.0281 (8)	0.0410 (12)	0.0477 (10)	0.0032 (7)	0.0011 (7)	−0.0013 (8)
C4	0.0289 (8)	0.0332 (10)	0.0383 (8)	−0.0017 (7)	−0.0016 (7)	−0.0022 (7)
C2	0.0450 (10)	0.0474 (13)	0.0429 (10)	0.0017 (9)	−0.0054 (8)	0.0043 (9)
C13	0.0381 (9)	0.0443 (12)	0.0423 (10)	0.0050 (8)	−0.0062 (8)	0.0023 (8)
C6	0.0378 (9)	0.0485 (13)	0.0459 (10)	−0.0095 (8)	0.0057 (8)	0.0001 (9)
C11	0.0377 (10)	0.0494 (13)	0.0516 (11)	−0.0049 (9)	0.0109 (8)	0.0016 (9)
C3	0.0288 (8)	0.0483 (13)	0.0482 (10)	0.0004 (8)	−0.0034 (7)	0.0044 (9)
C7	0.0528 (11)	0.0463 (13)	0.0392 (9)	−0.0045 (9)	0.0019 (8)	0.0038 (9)
C12	0.0516 (11)	0.0447 (13)	0.0408 (9)	−0.0021 (9)	0.0033 (9)	0.0053 (9)
C1	0.0560 (13)	0.085 (2)	0.0662 (14)	0.0045 (12)	−0.0112 (11)	0.0278 (14)

### Geometric parameters (Å, º)

**Table:**

N1—C8	1.301 (2)	C2—C3	1.367 (3)
N1—C9	1.404 (2)	C2—C7	1.400 (3)
N1—H1	0.895 (16)	C2—C1	1.508 (3)
C8—C4	1.410 (2)	C13—C12	1.377 (3)
C8—H8	0.9300	C13—H13	0.9300
O1—C5	1.304 (2)	C6—C7	1.369 (3)
C9—C10	1.391 (2)	C6—H6	0.9300
C9—C14	1.401 (2)	C11—C12	1.380 (3)
O2—C14	1.353 (2)	C11—H11	0.9300
O2—H4	0.932 (17)	C3—H3	0.9300
C14—C13	1.381 (3)	C7—H7	0.9300
C5—C6	1.410 (3)	C12—H12	0.9300
C5—C4	1.424 (2)	C1—H1A	0.9600
C10—C11	1.374 (3)	C1—H1B	0.9600
C10—H10	0.9300	C1—H1C	0.9600
C4—C3	1.407 (2)		
			
C8—N1—C9	127.59 (14)	C12—C13—C14	120.40 (17)
C8—N1—H1	113.3 (15)	C12—C13—H13	119.8
C9—N1—H1	119.1 (15)	C14—C13—H13	119.8
N1—C8—C4	123.26 (15)	C7—C6—C5	121.52 (17)
N1—C8—H8	118.4	C7—C6—H6	119.2
C4—C8—H8	118.4	C5—C6—H6	119.2
C10—C9—C14	119.50 (16)	C10—C11—C12	120.41 (17)
C10—C9—N1	123.55 (15)	C10—C11—H11	119.8
C14—C9—N1	116.95 (14)	C12—C11—H11	119.8
C14—O2—H4	111.4 (18)	C2—C3—C4	122.59 (17)
O2—C14—C13	123.12 (15)	C2—C3—H3	118.7
O2—C14—C9	117.36 (16)	C4—C3—H3	118.7
C13—C14—C9	119.52 (15)	C6—C7—C2	122.30 (18)
O1—C5—C6	122.24 (16)	C6—C7—H7	118.9
O1—C5—C4	121.25 (16)	C2—C7—H7	118.9
C6—C5—C4	116.51 (16)	C13—C12—C11	120.12 (18)
C11—C10—C9	120.02 (16)	C13—C12—H12	119.9
C11—C10—H10	120.0	C11—C12—H12	119.9
C9—C10—H10	120.0	C2—C1—H1A	109.5
C3—C4—C8	119.13 (15)	C2—C1—H1B	109.5
C3—C4—C5	119.91 (16)	H1A—C1—H1B	109.5
C8—C4—C5	120.96 (15)	C2—C1—H1C	109.5
C3—C2—C7	117.15 (17)	H1A—C1—H1C	109.5
C3—C2—C1	122.77 (18)	H1B—C1—H1C	109.5
C7—C2—C1	120.08 (18)		
			
C9—N1—C8—C4	179.90 (16)	O2—C14—C13—C12	178.77 (18)
C8—N1—C9—C10	8.4 (3)	C9—C14—C13—C12	−0.9 (3)
C8—N1—C9—C14	−171.45 (17)	O1—C5—C6—C7	178.47 (18)
C10—C9—C14—O2	−177.89 (16)	C4—C5—C6—C7	−1.0 (3)
N1—C9—C14—O2	2.0 (2)	C9—C10—C11—C12	−0.6 (3)
C10—C9—C14—C13	1.8 (3)	C7—C2—C3—C4	0.1 (3)
N1—C9—C14—C13	−178.31 (16)	C1—C2—C3—C4	179.5 (2)
C14—C9—C10—C11	−1.1 (3)	C8—C4—C3—C2	178.41 (19)
N1—C9—C10—C11	179.06 (17)	C5—C4—C3—C2	−1.3 (3)
N1—C8—C4—C3	−177.72 (17)	C5—C6—C7—C2	−0.2 (3)
N1—C8—C4—C5	2.0 (3)	C3—C2—C7—C6	0.7 (3)
O1—C5—C4—C3	−177.76 (17)	C1—C2—C7—C6	−178.8 (2)
C6—C5—C4—C3	1.7 (3)	C14—C13—C12—C11	−0.8 (3)
O1—C5—C4—C8	2.5 (3)	C10—C11—C12—C13	1.5 (3)
C6—C5—C4—C8	−177.99 (17)		

### Hydrogen-bond geometry (Å, º)

**Table:**

*D*—H···*A*	*D*—H	H···*A*	*D*···*A*	*D*—H···*A*
O2—H4···O1^i^	0.93 (2)	1.65 (2)	2.5756 (18)	176 (3)
N1—H1···O2	0.90 (2)	2.32 (2)	2.6598 (19)	102 (2)
N1—H1···O1	0.90 (2)	1.84 (2)	2.5933 (19)	141 (2)

Symmetry code: (i) −*x*+1, *y*+1/2, −*z*+1/2.
