# Supplementary figures and images for: Crystal structure of 2-{(E)-[(2-hy­droxy­phen­yl)iminium­yl]meth­yl}-4-methyl­phenolate
Source: Acta Crystallogr E Crystallogr Commun. 2015 Apr 9;71(Pt 5):o288. doi: 10.1107/S2056989015006374 (PMC4420074; doi:10.1107/S2056989015006374)

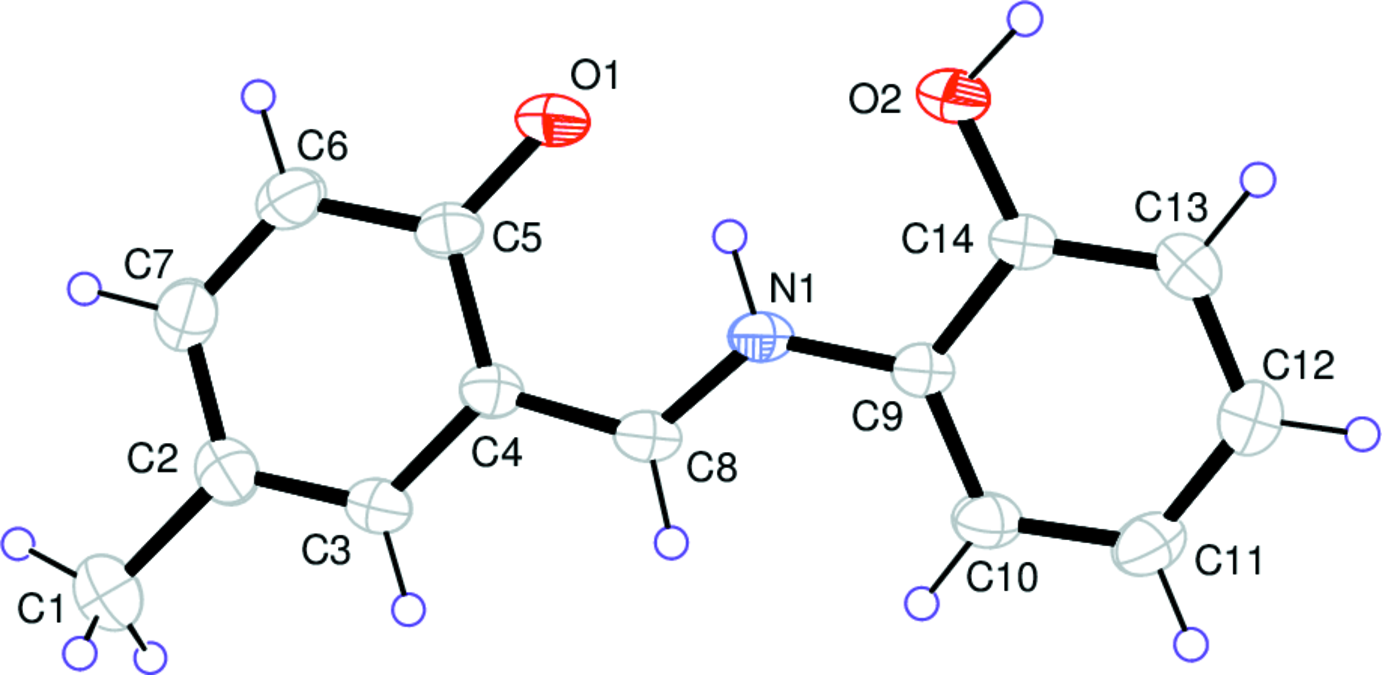

Supplement: Supplementary file 3 [file e-71-0o288-fig1.tif]

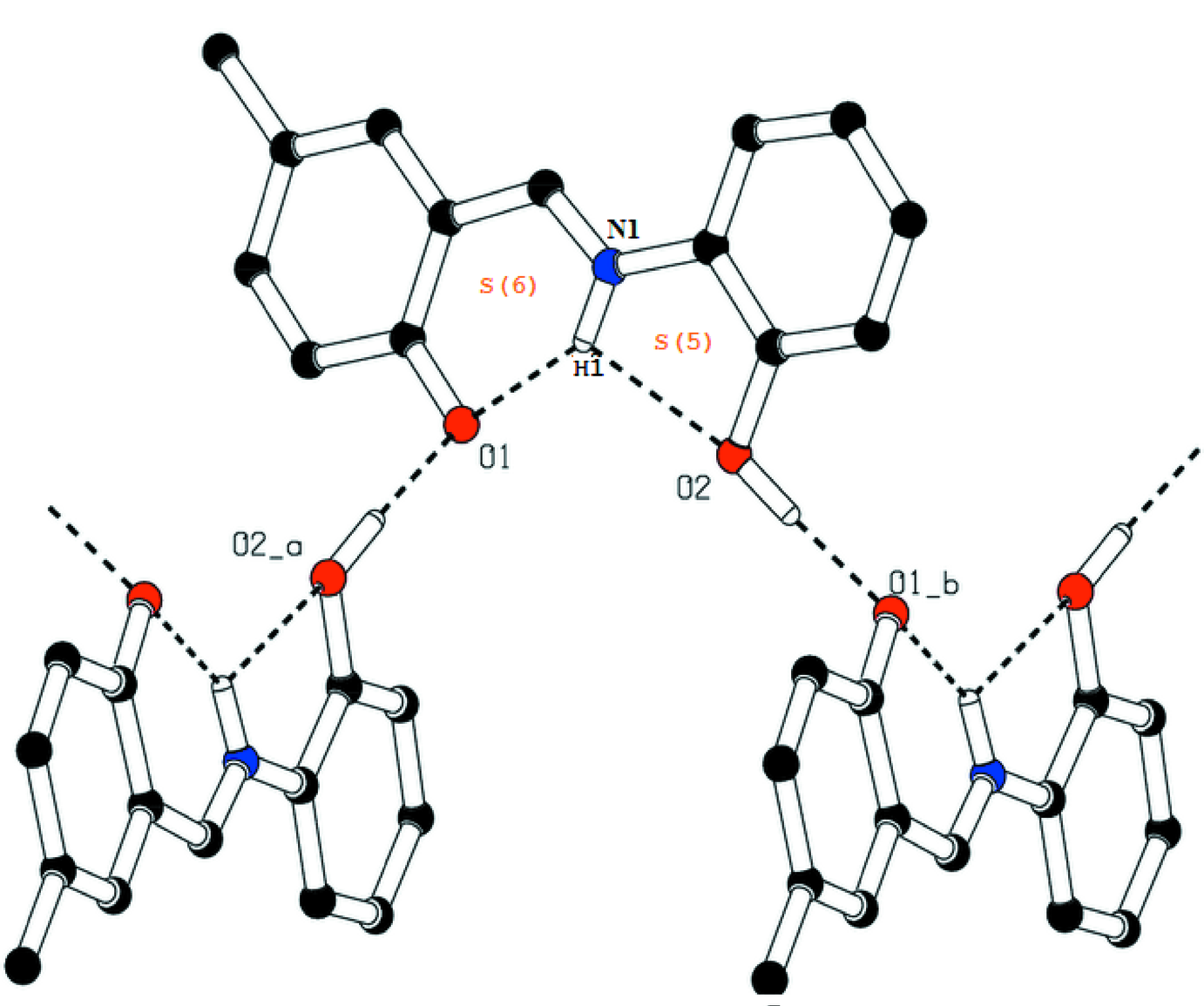

Supplement: Supplementary file 4 [file e-71-0o288-fig2.tif]
